# Investigating the genetic basis of susceptibility to amoebic gill disease and idiopathic gill lesions in Atlantic salmon populations using field data

**DOI:** 10.1186/s12711-025-01025-6

**Published:** 2026-01-22

**Authors:** Afees A. Ajasa, Solomon A. Boison, Muhammad L. Aslam, Marie Lillehammer, Hans M. Gjøen

**Affiliations:** 1 Nofima (Norwegian institute of Food, Fisheries and Aquaculture research), 210, N-1431 Ås, Norway; 2 Department of Animal and Aquacultural Sciences, Norwegian University of Life Sciences, 5003, N-1432 Ås, Norway; 3 Mowi Genetics AS, Sandviksboder 77AB, Bergen, Norway

## Abstract

**Background:**

Gill-related morbidity and mortality have become a major concern to the Atlantic salmon industry worldwide. Understanding the genetic mechanisms underlying susceptibility to gill diseases or lesions can help guide mitigation efforts. Genome-wide association analysis was conducted on gill scores from two large cohorts of Atlantic salmon populations, reared in Norway and Canada, that were phenotyped during amoebic gill disease (AGD) outbreaks and at harvest (referred to as idiopathic gill lesions (IGL)), respectively.

**Results:**

Whereas one novel quantitative trait locus (QTL) region on chromosome 12 was associated with susceptibility to AGD, two QTL regions on chromosomes 2 and 12 were associated with IGL. There was an overlap between the QTL region on chromosome 12 for AGD and IGL. The lead variant(s) identified explained approximately 7% of the additive genetic variance for AGD, and 3 and 10% for IGL, for the QTL on chromosomes 2 and 12, respectively. Putative candidate genes identified within or close to the lead variants include *tfeb*,* zscan12l*, and *ifi44l*, with the majority of these genes playing roles relating to immune functions. Fine-mapping the identified QTL region associated with AGD using re-sequence data revealed a lead intergenic variant explaining 9% of the additive genetic variance.

**Conclusions:**

Our results provide valuable insight into the genetic architecture of susceptibility to AGD and IGL, suggesting that both traits may be partly under the same genetic control. Future studies are warranted, especially on the genetic correlation between AGD and IGL.

**Supplementary Information:**

The online version contains supplementary material available at 10.1186/s12711-025-01025-6.

## Background

Gill-related diseases present a major challenge to the global Atlantic salmon (*Salmo salar*) aquaculture industry. In Norway for example, gill diseases are ranked among the top causes of mortality in Atlantic salmon farming [1]. Both infectious and non-infectious agents can cause gill diseases and lesions in Atlantic salmon [2]. Infectious agents include amoeba, bacteria, viruses, and others [2]. These agents can either act alone, for example, the amoeba *Parameoba (Syn. Neoparamoeba) perurans* causes amoebic gill disease (AGD) [3], or in concert, as in the case of complex gill disorder (CGD), where various disease agents co-occur [2, 4]. Non-infectious agents include harmful zooplankton and algae, and could also be a consequence of environmental and management activities, such as treatment of some infectious diseases [2, 5].

AGD significantly contributes to gill-related morbidity and mortality in Atlantic salmon [2]. It is a major concern in Norway, Scotland, Ireland, and Tasmania (Australia), and has also been reported in Chile and Canada [6]. Symptoms of this disease include inappetence, lethargy, flared opercula, abnormal swimming patterns, and death, if left untreated [7–9]. Grossly, AGD appears as white mucoid spots on the gills. Histologically, it is characterized by hyperplasia of the lamellar epithelium, hyperplasia and hypertrophy of mucous cells, and the presence of amoeba trophozoites [10]. The severity of AGD is usually assessed by subjective scoring of the gills based on Taylor’s scoring [11], and a freshwater or hydrogen peroxide bath is the only commercially utilized method of treating this disease [12, 13]. The expression of AGD is said to result from host resistance to the parasite (i.e. the parasite load on gills) and the localised host response to presence of the parasites (i.e. the extent of lesions) [14].

Recently, there has been a widespread increase in the occurrence of CGD [1, 4], making it a growing concern. Although the etiology of CGD is not fully understood, it is often associated with pathogens, including *Desmozoon lepeophtherii*, Candidatus Branchiomonas cysticola, and salmon gill pox virus (SGPV) [4]. Non-infectious agents may also contribute to the pathology of CGD [2, 4]. Symptoms of CGD include swollen and pale gills, inappetence, gill mucus accumulation, abnormal swimming behavior, respiratory distress, and significant mortality [4, 15]. Histopathologically, CGD is characterized by branchitis, epithelial cell hyperplasia, lamellar inflammation, necrosis, among other symptoms [16]. Cases where some or all of these symptoms are observed (gill damage) but where no specific known pathogen(s) can be attributed are also referred to as idiopathic gill lesions (IGL). Manifesting and recording of these disease phenotypes under experimental settings have proven to be challenging and an alternative is thus to use field data by ‘opportunistic harvesting of data’, which can take place during incidents of a disease epidemic or when the disease is endemic [17]. However, there are challenges associated with the use of field data for routine disease phenotyping, such as difficulty in ascertaining the real cause of mortality or morbidity, especially in sea cages, and unpredictability of disease outbreaks. Nevertheless, the use of field data is indispensable for some diseases due to welfare or health considerations [17], when the etiology of the disease is not well understood, or when the phenotype measured in the experimental or challenge settings is poorly genetically correlated with that obtained from the field, e.g., AGD [18, 19].

Ameliorating the negative consequences of AGD, CGD, and/or IGL requires a combination of different management strategies, for which selective breeding can be an effective tool. For example, in Tasmania, selective breeding for AGD resistance has helped to reduce the number of freshwater bath treatments required during the grow-out period, thereby reducing production costs [20, 21]. Resistance to AGD is now included in the breeding goal of most Atlantic salmon breeding programmes. However, faster selection response can be achieved with the knowledge of the underlying variants or gene(s) conferring resistance to AGD, CGD, and/or IGL. Genome-wide association (GWA) studies can help to unravel the genetic basis underlying susceptibility to AGD, CGD, and/or IGL, and this knowledge can help in the design of therapeutics [22], identify targets for gene edits, and for gene or marker-assisted selection [23]. While some GWA studies have been conducted on AGD [24–26], to the best of our knowledge, none has been conducted for CGD and/or IGL, likely due to the poor understanding of its etiology. In GWA studies, it is essential to confirm the detection of any QTL in an independent study or population [27, 28], however, GWA studies on AGD have so far not been consistent in their findings [24, 26, 29], and most of these studies [26, 29] fail to find a variant reaching genome-wide significance level. Several factors may be responsible for this: first, the genetic correlation between AGD measured in challenge trials and field settings is low [19], indicating that the traits measured in these environments are different; second, these studies differ in whether first or subsequent infections of AGD infections is studied, as Kube et al. [7] reported that the first infection or incidence of AGD is genetically different from subsequent re-infections; third, different quantitative trait loci (QTLs) may be segregating in different populations; fourth, the winners curse or Beavis effect [30, 31], i.e., the effects of significant single nucleotide polymorphisms (SNPs) may be overestimated; and finally, the trait could be highly polygenic.

Aquacultural breeding programmes often consist of multiple parallel populations at any point in time (see review by Gjedrem [32]). A good approach to increase sample size and consequently the power of detecting QTL is to combine these populations for GWA studies [33]. This approach also has the potential to increase mapping precision because across populations, linkage disequilibrium (LD) is only conserved at short distances [34, 35].

The breeding objective for AGD, CGD, and/or IGL and other gill diseases or lesions is improved gill health or resistance to gill diseases or lesions in the commercial production environment, which can reduce treatment requirements and improve fish welfare and performance. Hence, in this study, we aim to dissect the genetic architecture of AGD and IGL based on field data from large cohorts of Atlantic salmon, comprising several populations reared in Norway and Canada. We further refined the genomic region that was identified for AGD using sequence information.

## Methods

### Cohorts and study populations

The dataset used for this study was obtained from the breeding nucleus of an integrated salmon producer, Mowi ASA, in Norway and Canada, referred to as the Norwegian and Canadian cohorts, respectively. The Norwegian cohort has been described previously in Ajasa et al. [34, 36]. Briefly, the origin of the Norwegian cohort dates as far back as the sixties, when wild Atlantic salmon, mainly from the rivers Vosso and Åroy, were used to form four base populations [37] — in Atlantic salmon breeding programmes, four parallel populations often exist due to the four-year generation interval to provide eggs for the Atlantic salmon industry each year [32]. Between 1982 and 1986, ova from these populations were exported to Ireland to form the Mowi Fanad breeding population (s) [38]. A key attribute of Mowi’s Norwegian Atlantic salmon breeding programme before 2000 was the mixing of populations to avoid a rapid increase in inbreeding. Thus, the populations within the Norwegian cohort are to some degree admixed. In this study, 3 year-classes (YC, which is the year in which smolts were taken to sea) from Norway and 2 year-classes from Canada were included. One of the Norwegian year-classes included both Irish (Fanad) fish and fish with Norwegian origin, hence the Norwegian cohort consisted of four populations from three year-classes, namely: YC2016N, YC2016F, YC2017, and YC2018. In the 2016 YC, there was introgression with fish from Ireland (Fanad) to the Norwegian populations, hence the letters N and F are appended to the YC name to indicate Norwegian and Irish (Fanad) origin, respectively.

The Canadian cohort consisted of YCs 2017 and 2018. Each YC comprised 3 genetic populations, originating mainly from Mowi’s McConnell (M, of Scottish origin [39]), and Irish Fanad (F) strains. The letters M, F, and MF are appended to the population’s name to signify McConnell, Fanad, or a cross between both strains, respectively.

The Norwegian and Canadian cohorts used in this study are distantly related due to the early export of Mowi’s Fanad strains to Canada between 1991 and 1995. However, there has not been any recent introgression between the two independently run breeding programmes.

### Phenotypes

All populations within the Norwegian cohort were gill-scored based on Taylor’s scoring [11] during outbreaks of AGD. This involved scoring the fish from 0 to 5 based on the severity of AGD infection, with 0 indicating no infection and 5 indicating severe infection. The outbreaks of AGD occurred in the various years the populations were taken to sea, as indicated by the year class. Before AGD scoring, there was regular monitoring of fish gills. Once the presence of *Parameoba perurans* was confirmed by polymerase chain reaction (PCR), a random sample of anestheticized fish was scored weekly until the average gill scores reached 2, at which point all fish were gill-scored and treated with freshwater bath. The scoring was performed prior to the first AGD treatment of the fish after sea transfer.

For the Canadian cohort, the fish were gill-scored at harvest, also following Taylor’s scoring [11], i.e. 0–5, but solely on the percentage of lesion cover, as in Bloecher et al. [40]. Before gill scoring commenced, the fish were stunned with electrical charges, bled via the gills, and then kept in ice slurry. Veterinarians and health staff were unaware of any significant events that may have caused gill damage in the months prior to harvest. Unfortunately, no pathological samples were taken prior to or during the recording of this phenotype. Thus, the recorded gill lesions in the Canadian cohort will be referred to as IGL.

**Fig. 1:**
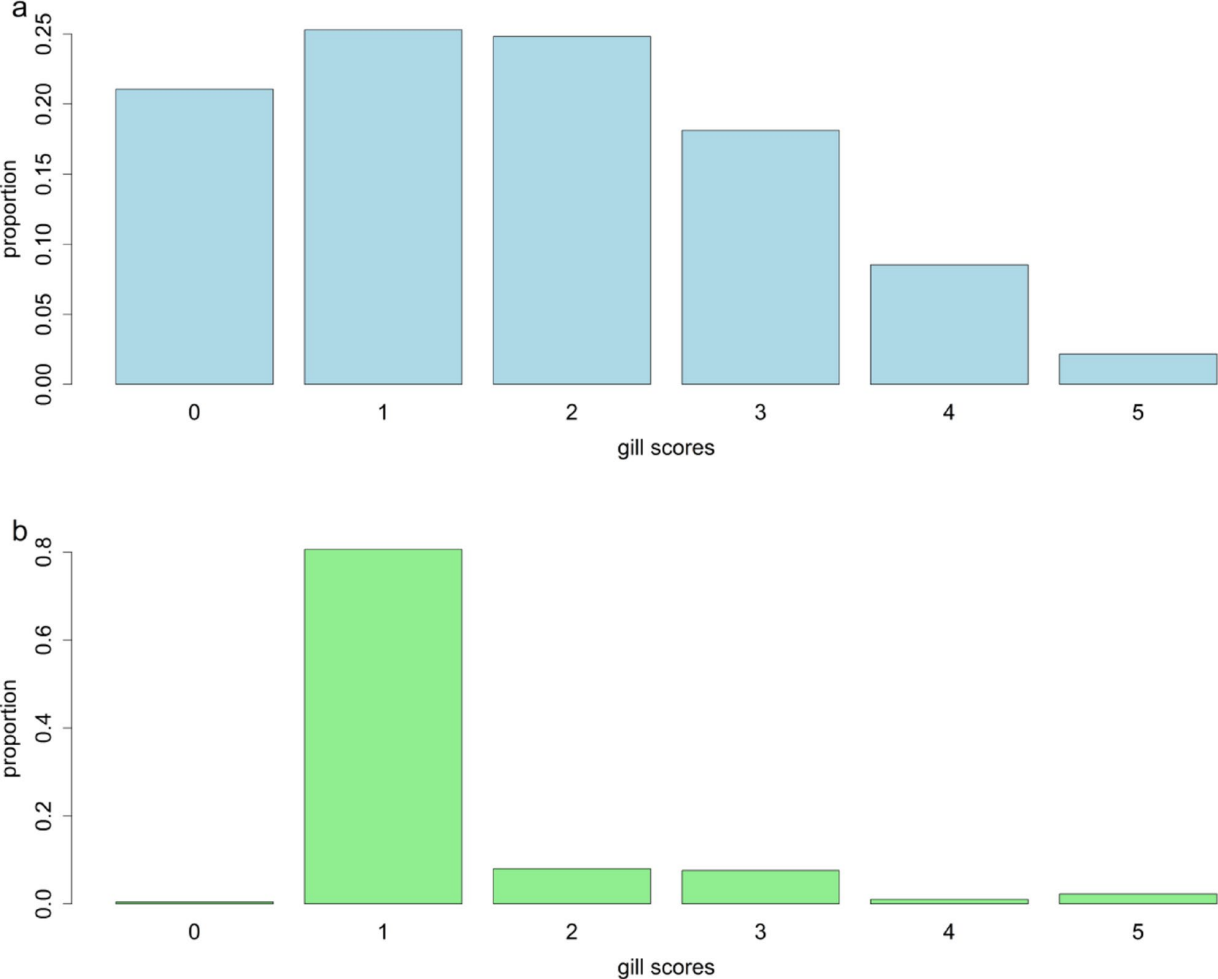
Bar plot of AGD gill scores (**a**) and IGL gill scores (**b**), for the Norwegian and Canadian cohort, respectively

**Fig. 2:**
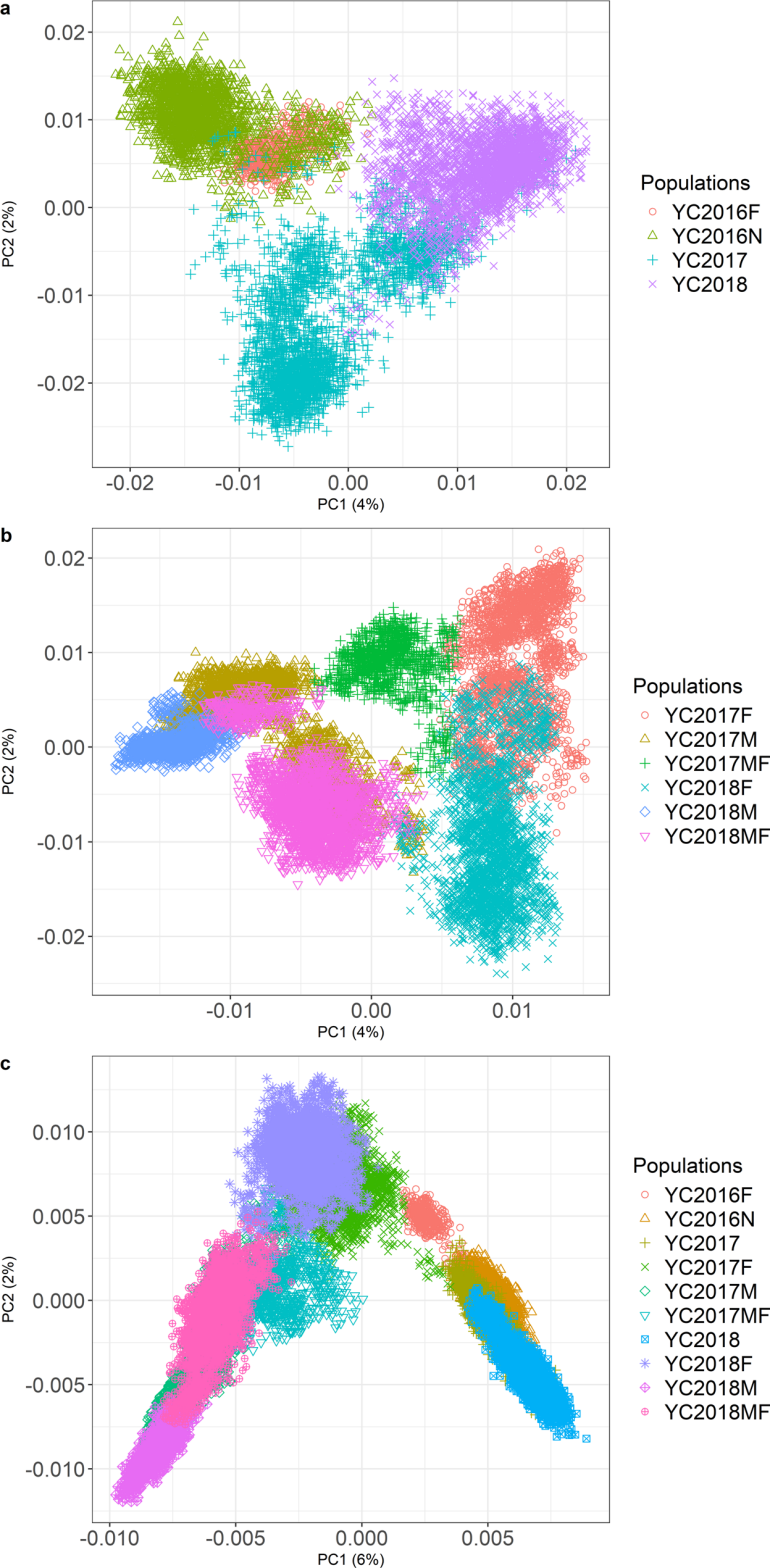
Principal components 1 and 2 for the Norwegian cohort (**a**), the Canadian cohort (**b**), and all individuals (**c**) in this study, constructed based on the respective genomic relationship matrices in GCTA [57]

Table 1 shows the number of animals per population for each cohort, as well as the descriptive statistics of the gill scores. The stocking date, month of recording, and average weight at gill scoring are also shown in Table 1. Figure 1a and b show the distribution of gill scores for AGD and IGL for the Norwegian and Canadian cohort, respectively. **Additional File 1**: Figures 1 and 2 show the distribution of gill scores for the various populations that were phenotyped for AGD and IGL, respectively.

**Table 1:** Descriptive statistics of gill scores per population for the Norwegian and Canadian cohorts

Cohort	Stocking date	Recording date	Population	Number of fish	Mean gill score (SD)	Median gill score	Range (min–max)	Mean weight (kg)
Norwegian^1^	15 Apr 2016	Nov 2016	YC2016N	2006	2.35 (1.35)	2	0–5	1.56
	15 Apr 2016	Nov 2016	YC2016F	640	1.91 (1.29)	2	0–5	1.04
	21 Apr 2017	Nov 2017	YC2017	2911	1.52 (1.31)	1	0–5	N/A
	19 Apr 2018	Nov 2018	YC2018	2949	1.52 (1.18)	1	0–5	2.49
Canadian^2^	23 Mar 2017	20–30 Aug 2018	YC2017M	2545	1.44 (0.93)	1	1–5	5.50
	23 Mar 2017	20–30 Aug 2018	YC2017F	2548	1.44 (0.98)	1	1–5	6.06
	23 Mar 2017	20–30 Aug 2018	YC2017MF	1125	1.47 (0.98)	1	1–5	5.86
	08 Feb 2018	20–23 Jul 2019	YC2018M	1425	1.29 (0.70)	1	0–5	6.04
	08 Feb 2018	20–23 Jul 2019	YC2018F	2544	1.23 (0.69)	1	0–5	5.86
	08 Feb 2018	20–23 Jul 2019	YC2018MF	2636	1.26 (0.76)	1	0–5	5.99

^1^ = AGD gill scores and ^2^ = IGL scores

### Genotypes

All fish were genotyped using a 55k SNP chip developed by Nofima in collaboration with Benchmark and Mowi Genetics. DNA extraction from fin clips and genotyping were performed by IdentiGEN Ltd. (https://identigen.com/; Dublin, Ireland). Quality control procedures for the Norwegian cohort were described by Ajasa et al. [36]. Briefly, samples and markers with a missing rate > 5% were removed. Markers with minor allele frequency (MAF) < 1% and Hardy-Weinberg p-value < 10e^− 25^ (Fisher’s exact test) were also excluded using Plink v 1.90b6.26 [41]. To further limit the impact of poor-quality samples, only samples with heterozygosity between 0.25 and 0.45 were kept [42]. Sporadic missing genotypes were imputed using Beagle [43]. These quality control procedures were also applied by YC for the Canadian cohort. Overall, the Norwegian cohort had 50,456 markers, while the Canadian cohort had 50,096 SNPs. Whereas the cohorts are genetically quite distinct from each other based on genetic relationships and genetic distance, in general no strong genetic differentiation existed within each of the cohorts (Fig. 2, see Additional file 2: Tables S1a and b). Because the whole genome sequence data (described below) were mapped to version 3 of the salmon genome (Ssal_v3.1), the positions of all SNPs for both cohorts were re-mapped from version 2 (ICSASG_v2) to the version 3.

### Sequence data variant calling

Whole genome sequencing was performed by BGI. Genomic DNA samples were used to prepare TruSeq PCR-Free whole-genome libraries, which were subsequently sequenced on the BGISEQ-500 platform, generating paired-end reads with a read length of 150 bp. The sequence reads were from 273 fish, which were mostly siblings of the Norwegian cohort. These reads were sequenced on four lanes, resulting in multiple sets of reads per sample. The quality of the reads was assessed using FASTQC [44] and low quality sequence segments were trimmed from the sequence data using Trimmomatic v 0.39 [45] with the following parameters: *LEADING:15* to remove bases with quality < 15; *TRAILING:15* to remove trailing bases with quality < 15; *SLIDINGWINDOW:4:15* to scan the reads using a 4-base wide sliding window and cut when average quality per base < 15; *MINLEN:36*, to drop reads < 36 bases long. The filtered high-quality reads were thereafter aligned to the latest version of the salmon genome (Ssal_v3.1) using Burrows-Wheeler Alignment’s (BWA) MEM- algorithm v 2.0 [46], and the resulting BAM files were sorted for the coordinates of aligned reads using SAMtools v 1.2 [47, 48]. Aligned reads of the same sample on different lanes were aggregated while concurrently marking duplicates using Picard’s v 3.0 [49] *MarkDuplicates*. Multi-sample variant calling was performed using HaplotypeCaller, GenomicsDBImport, and GenotypeGVCFs in GATK v 4.2.6.1 [50]. SNPs were extracted from the resulting VCF file with *SelectVariants* and *select-type SNP* function in GATK and filtered with *VariantFiltration* in GATK v 4.2.6.1 [50] using the following parameters: *QualByDepth (QD) < 2.0*,* QUAL < 30.0*,* StrandOddsRatio (SOR) > 3.0*,* FisherStrand (FS) > 60.0*,* RMSMappingQuality (MQ) < 40.0*,* MappingQualityRankSumTest (MQRankSum) < −12.5*,* ReadPosRankSumTest (ReadPosRankSum) < −8.0 (*https://gatk.broadinstitute.org/hc/en-us/articles/360035890471-Hard-filtering-germline-short-variants*).* All steps were performed following GATK best practices [50]. Variant calling was limited to chromosome 12, based on an initial GWA analysis result.

Processed sequenced data were also available from another 111 individuals from Mowi’s Norwegian breeding nucleus from another project (CMSEdit: NFR project no. 294504) and these were combined with the 273 individuals sequenced in the present study. Similar processing procedures were utilized for both datasets. Before merging these datasets with BCFtools v 1.10.2 [51], the VCF files were first zipped using Bgzip v 1.10.2 [52] and indexed using BCFtools v 1.10.2 [51]. SNPs were then extracted using *SelectVariants* and the *--exclude-filtered* option in GATK v 4.2.6.1 [50]. Biallelic SNPs were retrieved using VCFtools v 0.1.17 [53] with the options --*min-alleles 2* and *--max-alleles 2*. Markers with a MAF of 0.01, and an HWE p-value of < 10e^− 20^ were removed. To improve the quality of raw genotype calls and impute sporadic missing genotypes, Beagle v 4.1 [54] was used with the *-gl* option, as in Lloret-Villas et al. [55], resulting in 273,990 markers to be analyzed on chromosome 12. Thereafter, the genotypes were rephased using Beagle v 5.4 [43].

### Imputation

Based on an initial GWA analysis result on AGD using a medium-density array, the SNP genotypes on chromosome 12 of individuals from the Norwegian cohort were imputed to sequence level. As all sequenced individuals (384) only had genetic ties (sib relationships) with the Norwegian cohort, to avoid compromising imputation accuracy, genotype imputation could only be performed on individuals recorded for the AGD trait and not for the Canadian cohort. Imputation was done using Beagle v 5.4 [43] with default options. We excluded variants with imputation accuracy < 0.8, as derived from a 20-fold cross-validation implemented in R [56]. Each fold consisted of 5% of the sequenced individuals, randomly assigned as the validation set, and their sequence data were reduced to the variants available on the array data. The masked genotypes were then imputed using sequence information of the remaining 95% as reference animals. This process was repeated 20 times, and all individuals only appeared once in the validation set. Accuracy was calculated as the mean correlation between true genotypes and imputed genotypes. The number of SNP remaining after filtering SNPs with a MAF < 0.01 was 113,578.

### GWA analysis

GWA analysis was conducted separately on the Norwegian and Canadian cohorts. For each cohort, all populations were combined in a multi-population GWA analysis, using GCTA v 1.94.0 software [57] (*--mlma* option), while fitting the following model for each of the SNPs on the SNP chip genotype data:

$$\:\mathbf{y}=\mathbf{W}\mathbf{q}+\mathbf{x}b+\mathbf{Z}\mathbf{u}+\mathbf{e},$$

where **y** is the vector of gill scores, **W** is an incidence matrix relating the phenotype to the vector of fixed effects **q** (i.e. year for AGD; sex and production environment nested within year for IGL), **x** is the vector of genotypes for the evaluated SNP (coded 0|AA, 1|AG, 2|GG), *b* is the allele substitution effect, **Z** is an incidence matrix relating the phenotype to the vector of polygenic effects **u**, and **e** is the vector of residual effects, with distributional assumptions: **u** ~ N (0, **G**$\:{\sigma\:}_{\mathrm{u}}^{2}$) and **e** ~ N (0, **I**$\:{\sigma\:}_{\mathrm{e}}^{2}$), where **I** is an identity matrix, $\:{\sigma\:}_{\mathrm{e}}^{2}$ is the residual variance, **G** is the genomic relationship matrix (GRM) constructed based on default settings, and $\:{\sigma\:}_{\mathrm{u}}^{2}$ is the additive genomic variance. A Bonferroni threshold of 0.05/number of SNPs was used to determine the significance threshold, which was 9.91e^− 07^ and 9.98e^− 07^ for Norwegian and Canadian cohorts, respectively. The chromosome-wide significance threshold was calculated as 0.05 divided by the mean number of SNPs per chromosome. To investigate the influence of population structure or stratification on the GWA results, the genomic inflation factor (λ) was estimated as the median of the chi-square of the GWA test statistics divided by the median expected under a null distribution (0.455). The percentage of additive genetic variance (*pve*) explained by a SNP was estimated as:

$\:Pve=\frac{2p\left(1-p\right){b}^{2}}{{\sigma\:}_{\mathrm{u}}^{2}}\times\:100\%$, where *p* is the MAF, *b* is the estimate of the allele substitution effect, and $\:{\sigma\:}_{\mathrm{u}}^{2}$ is the estimate of the additive genomic variance. The estimates of $\:{\sigma\:}_{\mathrm{u}}^{2}$ and other variance components were obtained for the combined Norwegian or Canadian cohort using the above model without the fixed effect of the SNP, using GCTA v 1.94.0 (--reml option). Genetic correlations between the same trait (AGD or IGL) measured in different populations within cohort were estimated using Wombat [58], as in Ajasa et al. [34]. By default, Wombat uses the parameter expanded expectation maximisation (PX-EM) followed by the “average information” restricted maximum likelihood (AI REML) algorithm to estimate the variance component.

### Fine mapping, variant annotation, and haplotype analysis

Filtered sequence variants from chromosome 12 were analyzed using the same GWA analysis model as described for AGD. A Bonferroni threshold of 4.40e^− 07^, derived by dividing 0.05 by the number of sequence variants tested, was used to determine significance. Significant sequence variants were annotated using the Ensembl variant effect predictor [59]. We used locus Zoom R [60] to plot the significant chromosomal region, i.e. ±500 kb of the lead variant. Haplotype analysis was performed on all sequence data variants within the ± 500 kb window from the lead variants. Haplotype blocks were created for fixed 10 kb windows, and within each block, haplotypes with a frequency > 5% were converted to diplotypes (R script is available at [61]). Association analyses was conducted for these diplotypes using GCTA [57] and the same model as described for AGD, except that the fixed effect of diplotype was tested instead of SNP genotype. A Bonferroni threshold of 1.5e^− 04^ was used to determine the significance level of haplotypes, i.e. 0.05/number of tested haplotypes (326).

## Results

### Genetic parameters

Estimates of genetic parameters for AGD and IGL for the Norwegian and Canadian cohorts are shown in Table 2. The heritability estimates were around 0.2 for both traits. Within cohort estimates of heritability for each population are shown in Additional file 2: Table S14 and ranged from 0.14 to 0.25. Estimates of genetic correlation between the same trait measured in different populations within cohort are shown in Table 3, and ranged from an 0.22 to 0.99, with most estimates having high standard errors.

**Table 2:** Estimates of genetic parameters of AGD and IGL for the Norwegian and Canadian cohorts

Cohort	Trait	σ_u_^2^ ± SE	σ_e_^2^ ± SE	h^2^
Norwegian	AGD	0.36 ± 0.03	1.29 ± 0.02	0.22 ± 0.02
Canadian	IGL	0.14 ± 0.01	0.58 ± 0.01	0.20 ± 0.01

σ_u_^2^ = additive genomic variance, σ_e_^2^ = residual variance, SE = standard error, h^2^ = heritability

**Table 3:** Estimates of genetic correlation between populations, for AGD and IGL scores, within the Norwegian and Canadian cohort, respectively

Cohort and populations		Norwegian			Canadian				
		YC2016F	YC2017	YC2018	YC2017F	YC2017MF	YC2018M	YC2018F	YC2018MF
Norwegian^1^	YC2016N	0.91 ± 0.51	0.99 ± 0.23	0.65 ± 0.26	–	–	–	–	–
	YC2016F	–	0.99 ± 0.48	0.98 ± 0.57	–	–	–	–	–
	YC2017	–	–	0.95 ± 0.16	–	–	–	–	–
Canadian^2^	YC2017M	–	–	–	0.90 ± 0.40	0.99 ± 0.29	0.99*	0.75 ± 0.32	0.91 ± 0.19
	YC2017F	–	–	–	–	0.99 ± 0.35	0.99*	0.94 ± 0.21	0.99 ± 0.36
	YC2017MF	–	–	–	–	–	0.99 ± 0.68	0.21 ± 0.43	0.64 ± 0.36
	YC2018M	–	–	–	–	–	–	0.99 ± 0.55	0.94 ± 0.18
	YC2018F	–	–	–	–	–	–	–	0.99 ± 0.21

*Convergence was achieved with the Expectation Maximisation (EM) algorithm in the Wombat software [58], which does not provide standard errors of the estimates. The GRM for estimating the genetic correlation was based on Zhou et al. [62] and Wientjes et al. [63]

^1^ = AGD gill scores and ^2^ = IGL scores

### GWA analysis

Unless stated otherwise, the results described below are based on analysis of the combined populations within cohorts. The estimated genomic inflation factor, λ, were 0.96 and 0.94 for AGD and IGL, respectively, indicating that there was no inflation of our test statistics by population structure or stratification.

**Fig. 3:**
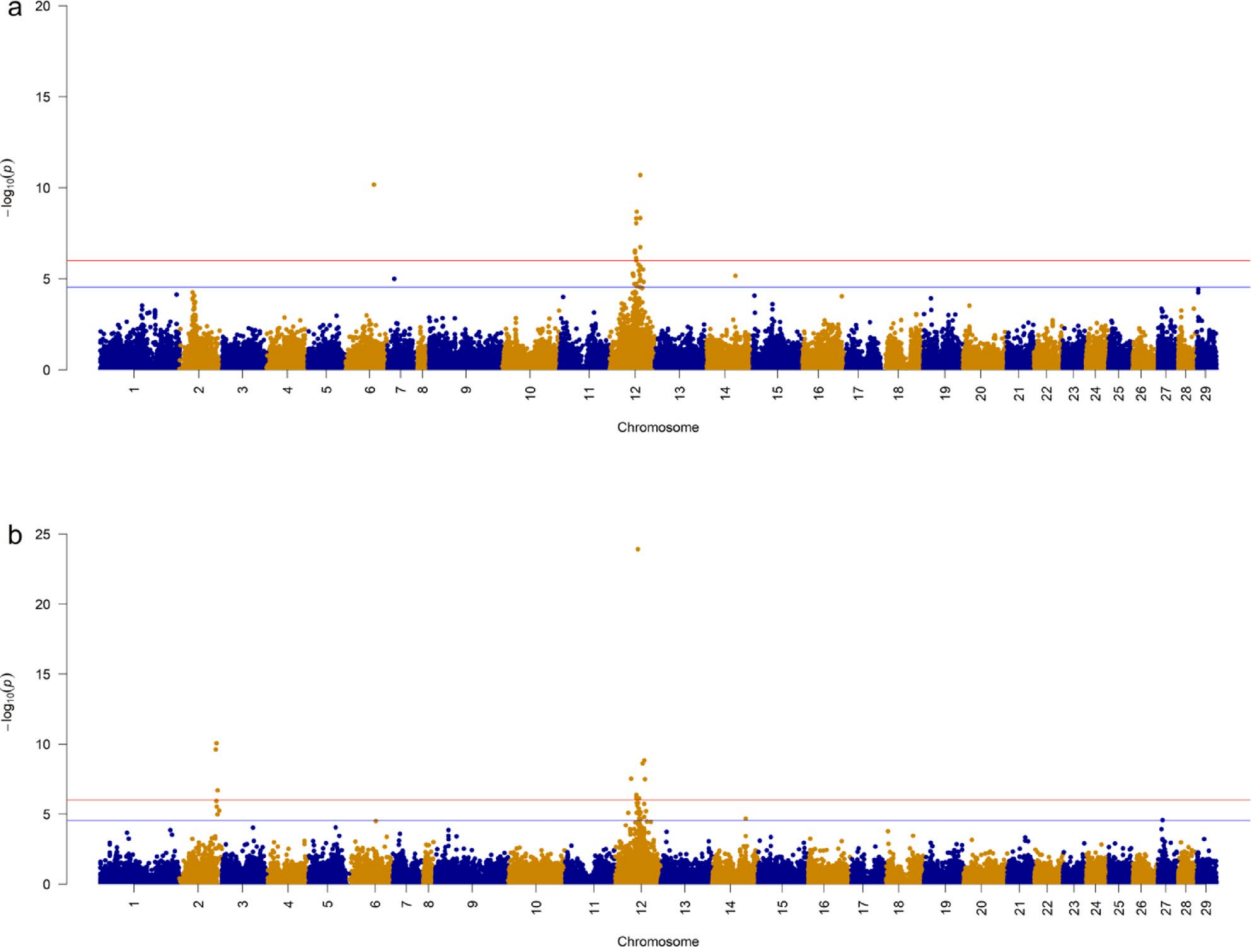
Manhattan plot of summary statistics derived from genome-wide association analysis of AGD (**a**) and IGL (**b**). Legend: The red line indicates the Bonferroni threshold while the blue line indicates the chromosome-wide significance threshold

The GWA analysis identified one significant QTL region on chromosome 12 for AGD (Fig. 3a) and two other regions, on chromosomes 2 and 12, were significantly associated with IGL (Fig. 3b). The summary statistics for the lead SNP in the identified regions for both traits, as well as the closest gene(s), are shown in Table 4. Although the QTL regions on chromosome 12 for AGD and IGL overlapped, their lead SNPs were different (Table 4). The percentage of additive genetic variance explained by the lead variant(s) was ~ 7% for AGD and 3 and 10% for IGL, for the QTL on chromosomes 2 and 12, respectively. Based on the salmon genome database (Ssal_v3.1- GenBank accession GCA_905237065.1), the closest candidate gene(s) to the lead variant(s) are also shown in Table 4 and included *tfeb* (transcription factor EB) for AGD and *zscan12l* (Zinc finger and SCAN domain-containing protein 12-like), and *ifi44l* (interferon-induced protein 44-like) for IGL. The lone significant SNP on chromosome 6 for AGD is likely a false positive due to a misplaced marker during transformation to version 3 of the salmon genome, as it did not appear in the GWA analysis based on the original genotype data mapped to version 2. Additional file 2: Tables S2 and S3 present summary statistics of variants associated with AGD and IGL, respectively, while Manhattan plots and summary statistics of variants from GWA analysis for populations within cohorts are shown in Additional file 3: Fig. S1–S10, and Additional file 2: Tables S4–S13, respectively. Within populations, only one QTL region on chromosome 12 was found to be significantly associated with AGD resistance, whereas three QTL regions on chromosomes 2, 7 and 12, were found to be significantly associated with IGL.

**Table 4:** Lead variants for genomic regions associated with AGD and IGL based on the SNP chip genotype data

Trait	CHR	SNP	Mb	Freq	b	SE	*P*	$\:\boldsymbol{P}\boldsymbol{v}\boldsymbol{e}\:\left(\boldsymbol{\%}\right)$	(nearest) Gene
AGD	12	AX-98,319,206	69.36	0.22	0.26	0.04	2.04e^− 11^	6.68	*tfeb*
IGL	2	AX-96,373,699	82.60	0.15	0.13	0.02	8.72e^− 11^	2.96	*zscan12l*
	12	AX-87,970,497	51.69	0.33	0.18	0.02	1.22e^− 24^	10.25	*ifi44l*

CHR = chromosome, Mb = mega base pairs, Freq = effect allele frequency, SE = standard error, *Pve* = percentage of additive genetic variance explained

### Fine mapping, variant annotation, and haplotype analysis of the QTL region associated with AGD

**Fig. 4:**
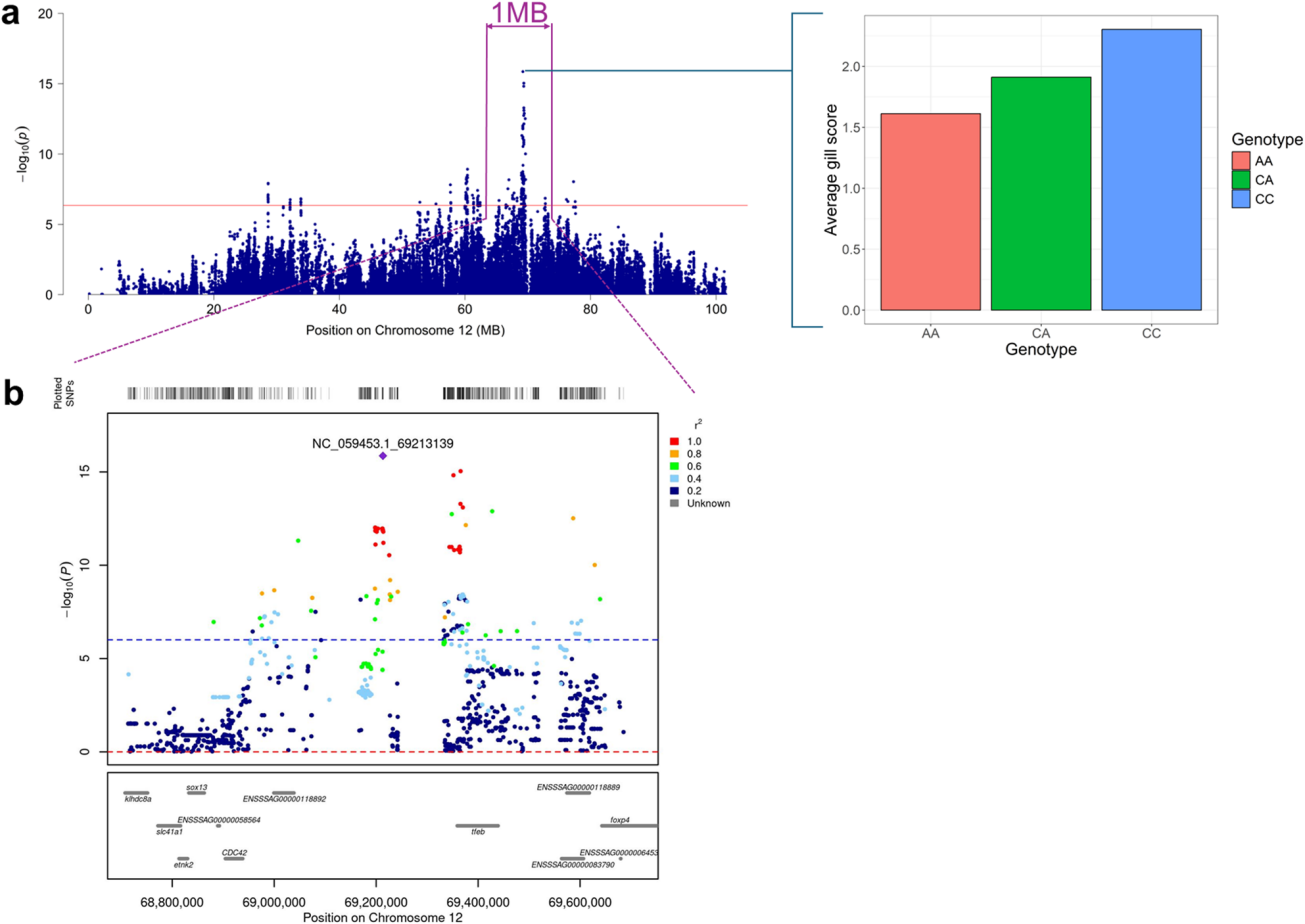
Manhattan plot of chromosome 12 based on sequence GWA analysis for AGD (**a**). The red line indicates the Bonferroni significance threshold, and the bar plot to its right is the average gill score for the lead variant genotypes. Locus Zoom plot 500 kb upstream and downstream of the lead SNP (**b**). The lead SNP is shown with a purple diamond. SNPs are colored based on their LD with the lead SNP, and the blue dashed line indicates the Bonferroni significance threshold. The genes within the plotted region are shown in the bottom pane

**Fig. 5:**
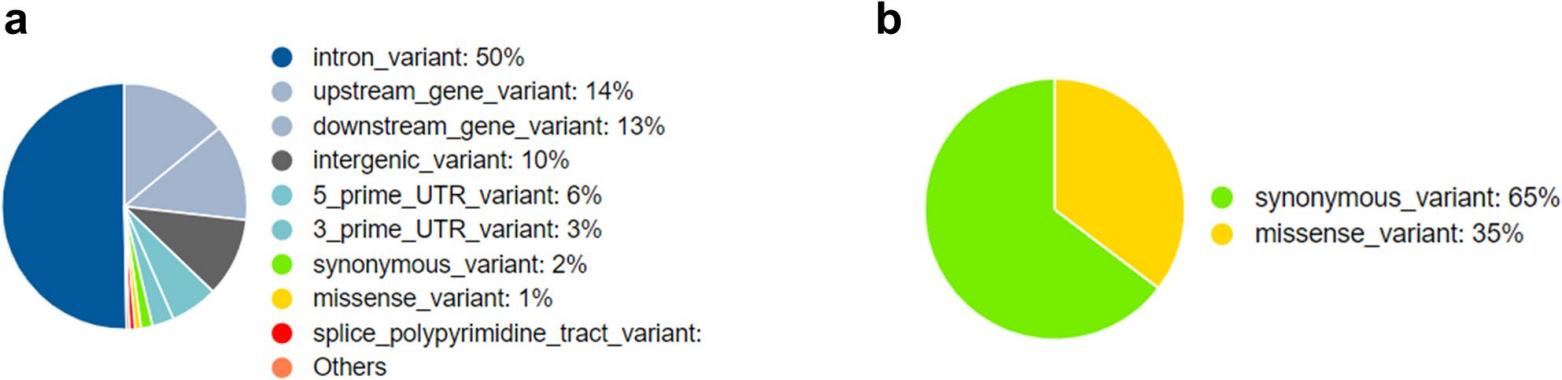
Summary of annotations of significant sequence variants (**a**). Annotation of significantly associated coding sequence variants (**b**).

To increase the resolution of the identified region on chromosome 12 for AGD, sequence information was analyzed and 251 SNPs were found to be significantly associated with susceptibility to AGD. The Manhattan plot of the sequence-based GWA analysis is shown in Fig. 4a. The lead SNP, NC_059453.1_69213139, with base pair position 69,213,139, explained 9% of the additive genetic variance for AGD (Fig. 4a, b and see Additional file 2: Table S15), and its genotypes AA, CA and CC had average gill scores of 1.61, 1.91 and 2.30, respectively. Variants annotation revealed that 50% of the significant variants were intronic variants (Fig. 5a), and most (96%) had a modifier impact, while a few had moderate (1.6%) or low (2.4%) impact (see Additional file 2: Table S16). The lead SNP is an intergenic variant (Fig. 4b) with modifier impact, close to the *tfeb* gene. Only a small fraction of variants were predicted to code for an amino acid and most of these did not result in a change of amino acids (Fig. 5b).

Significant haplotypes for the QTL on chromosome 12 are shown in Additional file 2: Table S17. The most significant haplotype had 11 SNPs and spanned from the region from 69.20 to 69.21 Mb. This haplotype had the same frequency and effect size as the lead sequence variant.

## Discussion

To gain insight into the genetic architecture of AGD and IGL, GWA analyses were conducted on two large cohorts of Atlantic salmon reared in Norway and Canada, respectively. We report novel QTL regions for both traits.

Unlike in most livestock species, numerous medium to large effect QTLs for complex traits, such as disease resistance, have been discovered in aquaculture species (see review by Yanez et al. [64]). Most remarkable is the case of the infectious pancreatic necrosis (IPN) virus, where a QTL explaining about 80% of the additive genetic variance has been discovered [65, 66], and its use for marker-assisted selection has helped to reduce IPN-associated mortality drastically [67]. This, among other reasons, has motivated continued interest in the GWA studies of complex traits in aquaculture populations.

As laid out in the Methods section, while the Norwegian cohort was gill-scored during AGD outbreaks, the Canadian cohort was gill-scored at harvest. The reason for combining all populations in a separate GWA analysis for each cohort was to increase the power of detecting QTL. Moreover, there was in general a high genetic correlation between the traits measured in each cohort (Table 3), suggesting the same traits were measured across year classes. Since LD across populations is only conserved at short distances [34, 35], each cohort’s GWA analysis should help refine the QTL position. Hence, the identified SNPs should be close to the causal gene, providing strong support for the candidate genes identified in this study. The detected lead SNP (AX-98319206) associated with susceptibility to AGD is a 5′UTR variant (Chr12:69,364,333) within the *tfeb* gene (Chr12: 69,359,094 − 69,439,303) and functions mainly to modulate the expression of other genes, such as autophagy and lysosomal biogenesis-related genes [68, 69]. The modulated genes perform functions related to lysosomal biogenesis and exocytosis, autophagy, lipid catabolism, energy metabolism, and immune response [70, 71]. During infection, *tfeb* drives the expression of genes related to host tolerance [72]. For AGD, the role of *tfeb* could be through its induction of cytokines and chemokines during infection [72]. Indeed, studies have shown differential expression of cytokines during AGD (see review by Marcos-Lopez and Rodger [10]). In addition, the *tfeb* gene has also been identified as a putative candidate gene for resistance to cardiomyopathy syndrome (CMS) in Atlantic salmon [73, 74]. Of the candidate genes identified within or close to the lead variants associated with IGL, only *ifi44l* (Chr12: 51,659,424 − 51,708,382) has a known function related to immune response [75], e.g., as reported through its role in response to viral infections in mice [75] and oysters [76]. *ZSCAN12l* (Chr02: 82,595,827 − 82,606,769), on the other hand, is an uncharacterized gene, whereas its homolog, *ZSCAN12*, is associated with cancer [77].

Unlike in previous studies [24, 26, 29], we detected the same QTL region (chromosome 12) for both traits in the different populations of the studied cohorts (see Additional file 2: Tables S4–S13 and Additional file 3: Fig. S1–S10). However, some QTL regions were detected only within populations, for example on chromosome 7 in YC2017MF for IGL (see Additional file 3: Fig. S7) and not in the joint analysis. This might be due to poor persistency of LD phase across populations, Beavis effect, or non-additive genetic effects. Conversely, some QTL regions were detected in the joint analysis but not within populations, which could be due to increased power from a larger sample size.

In this study, we report for the first time the heritability of susceptibility to IGL, which was about 0.2 for the corresponding cohort and most of the populations studied (Table 2 and see Additional file 2: Table S14), indicating that this trait can be improved by selective breeding. This estimate was similar to that obtained for susceptibility to AGD (see Additional file 2: Table S14). Higher [7] or lower [19] heritability estimates for AGD compared to what we found here have also been reported, which could be due to different QTL segregating in different populations, differences in severity of AGD in the studied population(s), inherent subjectivity of the gill scoring method, other compounding gill pathologies, size of fish [78] and differences in AGD infection (first vs. subsequent infections) [79]. Because the IGL phenotype was recorded at harvest, highly susceptible fish would have died early on; thus, the heritability estimates may be underestimated. Additionally, the IGL phenotype is likely reflective of a fish’s ability to cope with a variety or combination of infectious and non-infectious disease agents, which could be in the form of multifactorial gill disease or complex gill disease during its productive life cycle. Although the different genetic mechanisms involved in a fish’s response to AGD in challenge and field trials [18, 19] suggest that fish might respond differently to different gill infestations or diseases, for practical breeding purposes it is of interest to know the genetic correlation between AGD phenotype and IGL, especially in Norway where multifactorial gill diseases are becoming rampant. Hence, further studies are needed on the genetic correlation between AGD and IGL phenotypes using for example a split-family design [80], where sibs are recorded for both traits.

Fine mapping the genomic region associated with AGD resulted in a lead variant (NC_059453.1_69213139) that explained a higher percentage of additive genetic variance compared to the SNP chip lead variant, and its annotation suggests a regulatory role in AGD susceptibility. Gene-based analysis [81] of the sequence GWA analysis summary statistics also reveals *tfeb* as the most promising candidate gene (see Additional file 1: Table S18).

In principle, sequence data should contain causal variants; however, this might not be the case due to the coverage of sequence data, quality filtering, etc. Even if the causal variants were contained in the sequence data, other variants in LD with the causative variant(s) might still show a higher level of significance [82] as a result of Beavis effect [30, 31] or synthetic associations [83]. Hence, the identified lead SNPs in this study might not be the causal variant. Functional data involving transcriptomics and/or proteomic information together with functional validation, are thus necessary to further strengthen the current findings. Moreover, the most significant haplotype has the same frequency and effect as the lead variant, with positions ranging from 69,203,642 to 69,213,414 bp (intergenic region), which may indicate that a common haplotype affecting susceptibility to AGD is segregating across populations, further complicating the identification of the causal variants.

## Conclusions

GWA analysis revealed novel loci associated with susceptibility to AGD and IGL, with the lead variants explaining between 3 and 10% of additive genetic variance. Additionally, candidate genes were identified, thus enhancing our knowledge of the likely genetic mechanisms underlying AGD and IGL resistance. Our results also show that some regions might be associated with both AGD and IGL; however, further studies are warranted, particularly on the genetic correlation between AGD and IGL.

## Supplementary Information

Below is the link to the electronic supplementary material.

Supplementary Material 1

Supplementary Material 3

Supplementary Material 2

## Data Availability

The data used in this study were provided by Mowi Genetics AS and are not publicly accessible but can be made available upon reasonable request.
