# Supplementary material for: Investigating the genetic basis of susceptibility to amoebic gill disease and idiopathic gill lesions in Atlantic salmon populations using field data
Source: Genet Sel Evol. 2026 Jan 22;58:10. doi: 10.1186/s12711-025-01025-6 (PMC12857072; doi:10.1186/s12711-025-01025-6)
Supplement: Supplementary file 1 — Supplementary Material 1 [file 12711_2025_1025_MOESM1_ESM.docx]

**Investigating the genetic basis of susceptibility to amoebic gill disease and gill lesions in Atlantic salmon populations using field data**

*Afees A. Ajasa^1,2*^, Solomon A. Boison^3^, Muhammad L. Aslam^1^, Marie Lillehammer^1^ and Hans M. Gjøen^2^*

*^1^Nofima (Norwegian institute of Food, Fisheries and Aquaculture research), PO Box 210, N-1431 Ås, Norway*

*^2^Department of Animal and Aquacultural Sciences, Norwegian University of Life Sciences, 5003 NMBU, N-1432 Ås, Norway*

*^3^Mowi Genetics AS, Sandviksboder 77AB, Bergen, Norway*

**

**

**Figure S1: A bar plot of the various AGD gill scores categories for YC2016N (a), YC2016F (b), YC2017 (c) and YC2018 (d)**

**

**

**Figure S2: A bar plot of the various IGL gill scores categories for YC2017M (a), YC2017F (b), YC2017MF (c), YC2018M (d), YC2018F (e), and YC2018MF (f)**
