# Supplementary material for: Investigating the genetic basis of susceptibility to amoebic gill disease and idiopathic gill lesions in Atlantic salmon populations using field data
Source: Genet Sel Evol. 2026 Jan 22;58:10. doi: 10.1186/s12711-025-01025-6 (PMC12857072; doi:10.1186/s12711-025-01025-6)
Supplement: Supplementary file 2 — Supplementary Material 3 [file 12711_2025_1025_MOESM4_ESM.docx]

**Investigating the genetic basis of susceptibility to amoebic gill disease and gill lesions in Atlantic salmon populations using field data**

*Afees A. Ajasa^1,2*^, Solomon A. Boison^3^, Muhammad L. Aslam^1^, Marie Lillehammer^1^ and Hans M. Gjøen^2^*

*^1^Nofima (Norwegian institute of Food, Fisheries and Aquaculture research), PO Box 210, N-1431 Ås, Norway*

*^2^Department of Animal and Aquacultural Sciences, Norwegian University of Life Sciences, 5003 NMBU, N-1432 Ås, Norway*

*^3^Mowi Genetics AS, Sandviksboder 77AB, Bergen, Norway*

**Manhattan plots for different populations recorded for AGD**


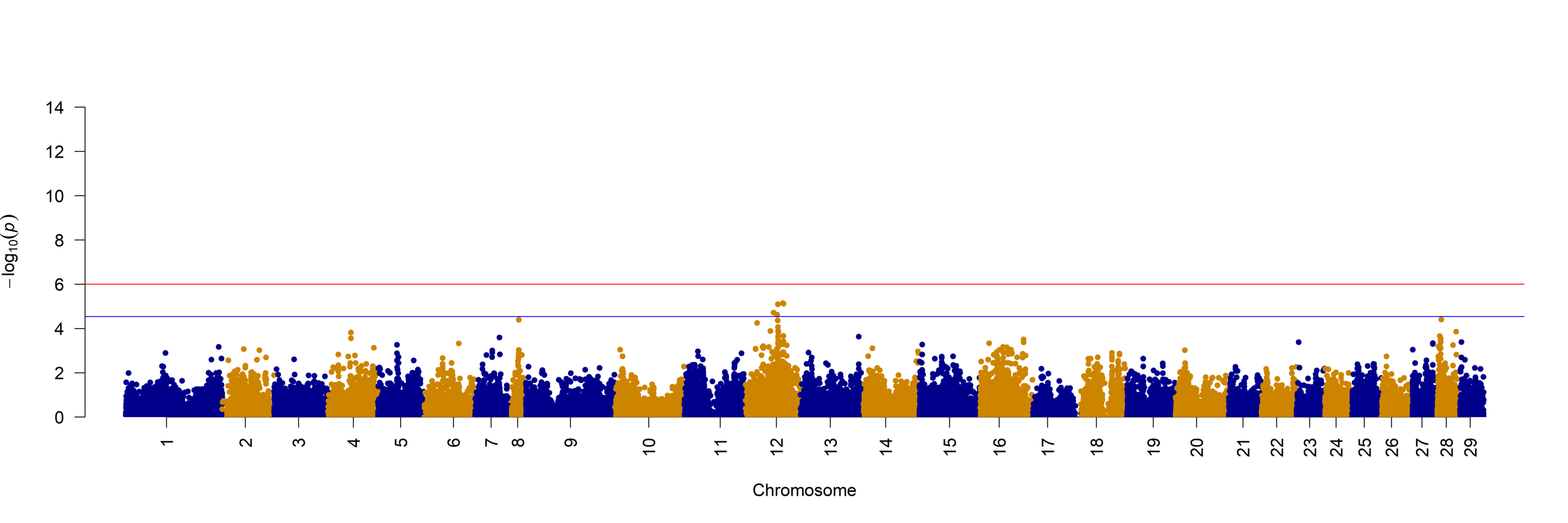


**Figure S1:** Manhattan plot of summary statistics derived from genome-wide association analysis of AGD for YC2016N.


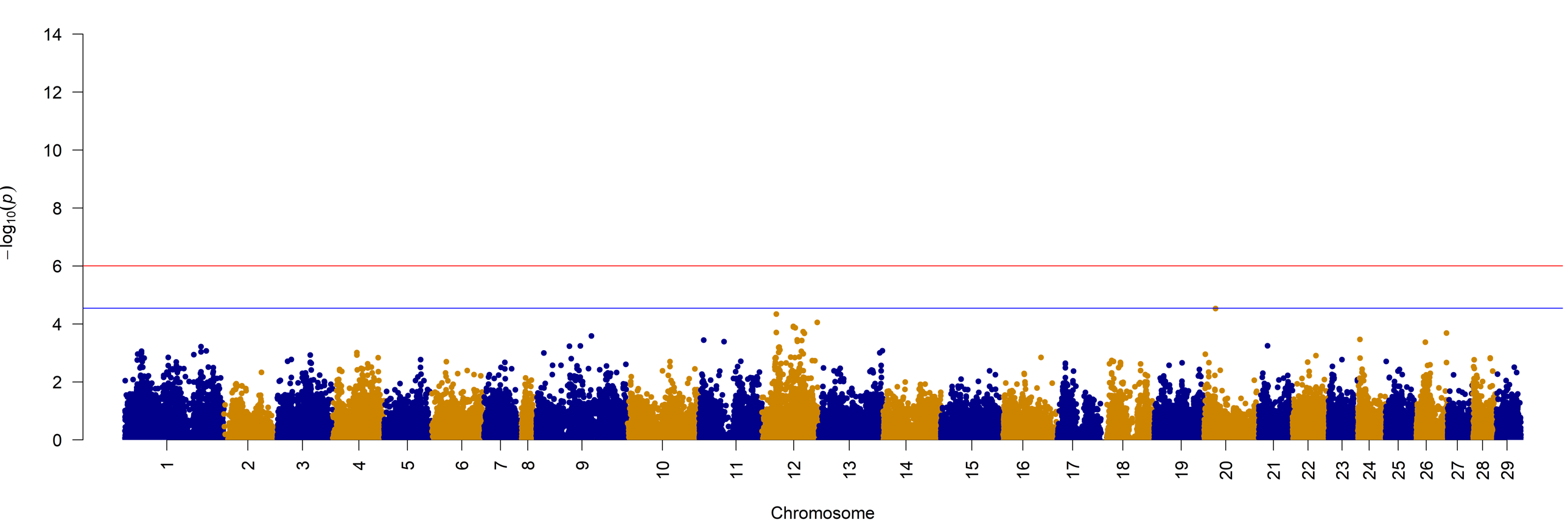


**Figure S2:** Manhattan plot of summary statistics derived from genome-wide association analysis of AGD for YC2016F.


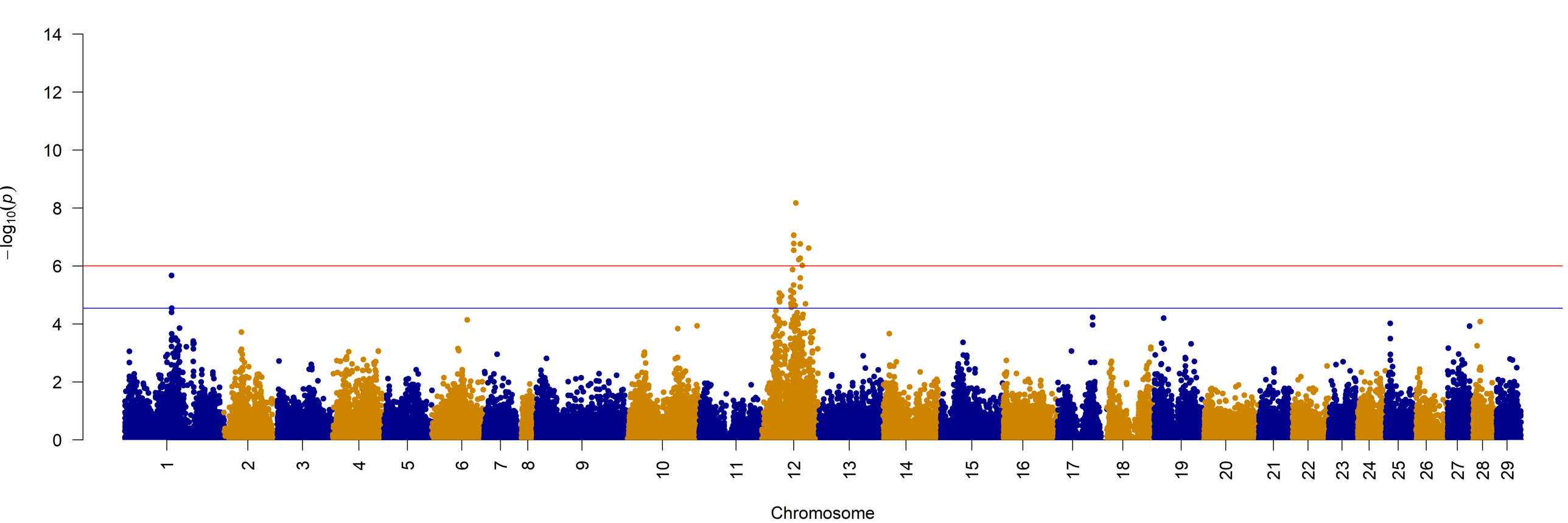


**Figure S3:** Manhattan plot of summary statistics derived from genome-wide association analysis of AGD for YC2017.


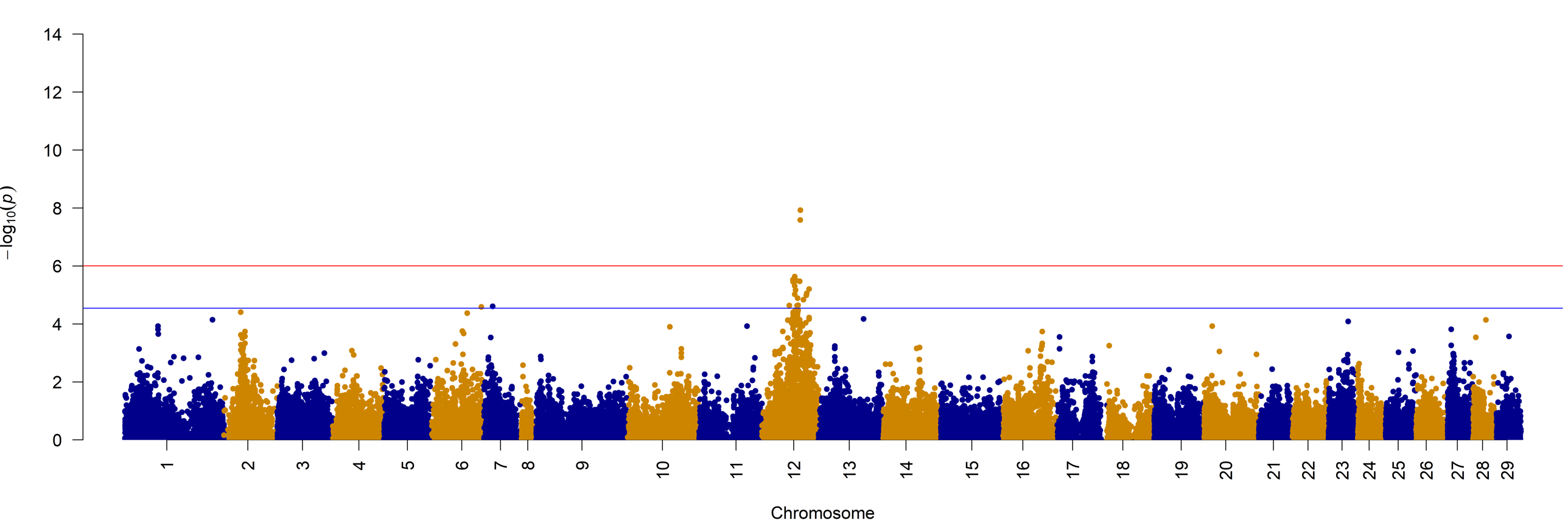


**Figure S4:** Manhattan plot of summary statistics derived from genome-wide association analysis of AGD for YC2018.

**Manhattan plots for different populations recorded for Idiopathic Gill lesions**


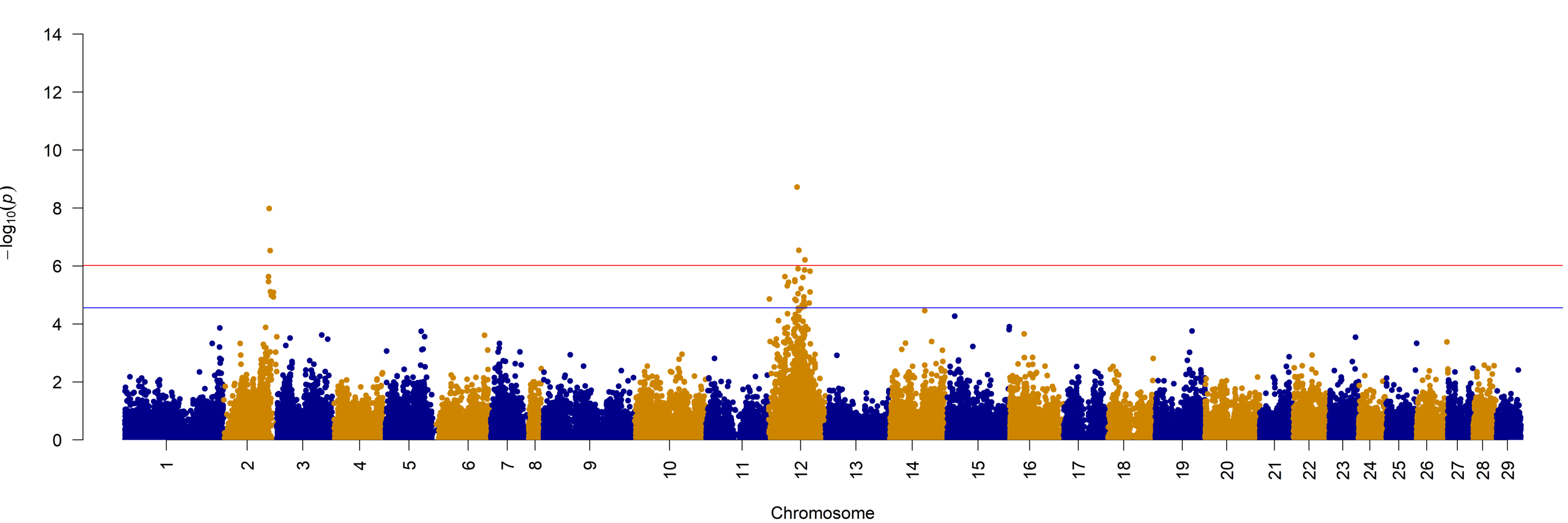


**Figure S5:** Manhattan plot of summary statistics derived from genome-wide association analysis of IGL for YC2017M.


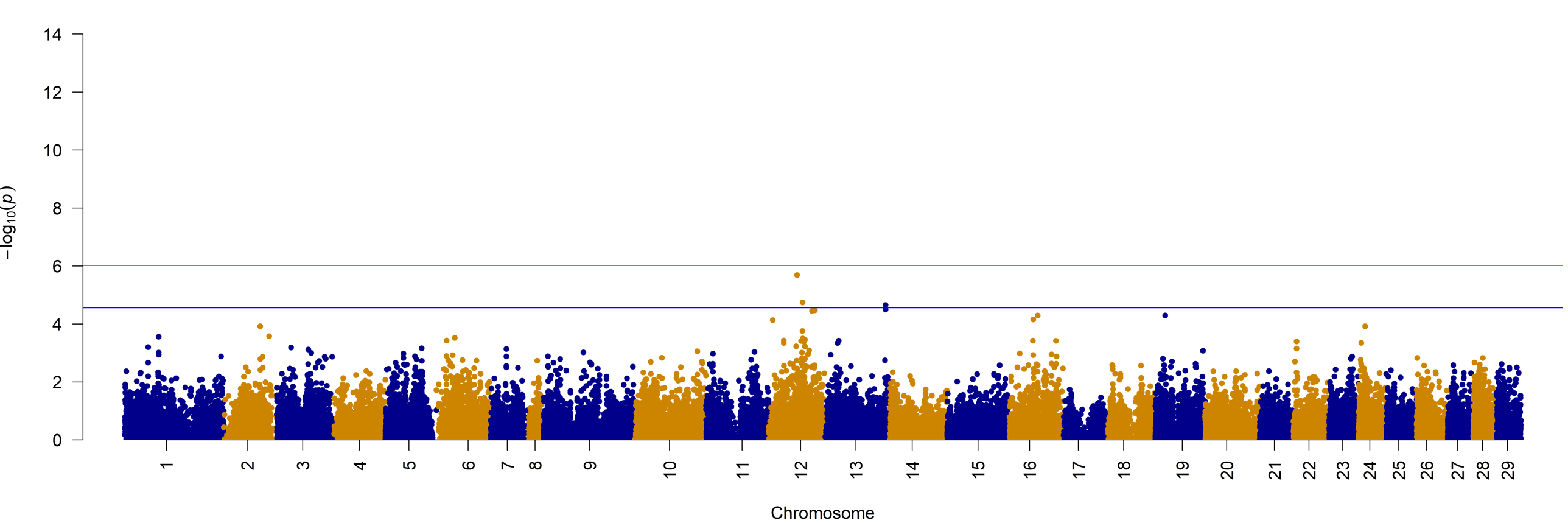


**Figure S6:** Manhattan plot of summary statistics derived from genome-wide association analysis of IGL for YC2017F.


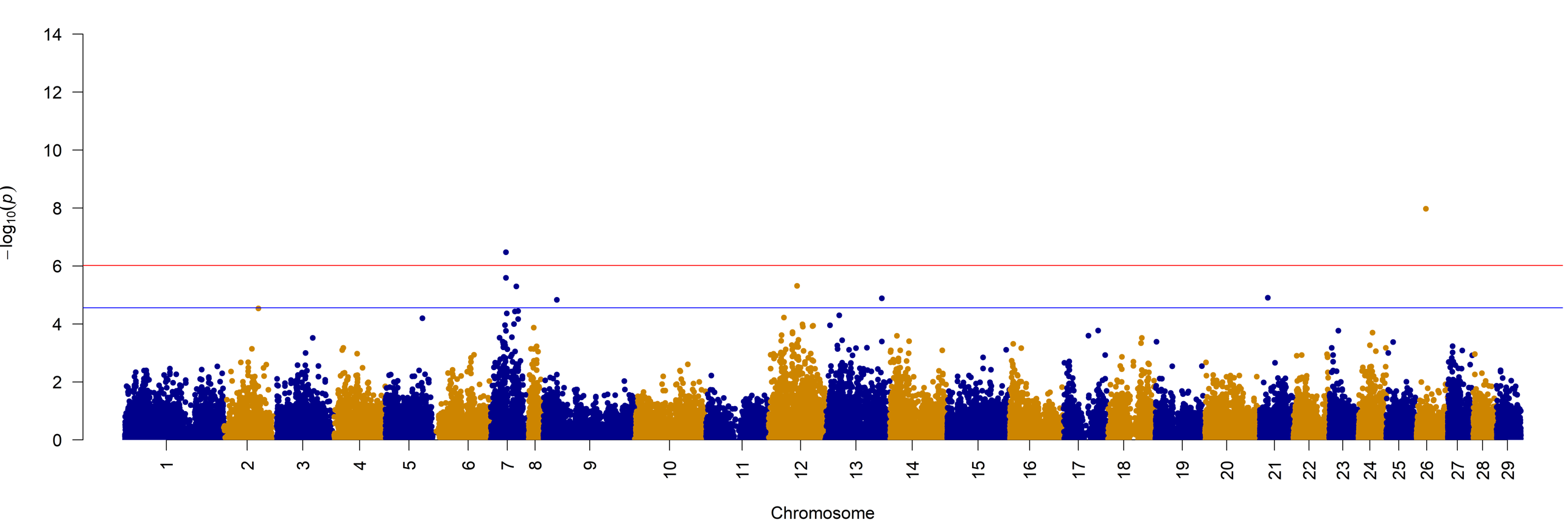


**Figure S7:** Manhattan plot of summary statistics derived from genome-wide association analysis of IGL for YC2017MF.


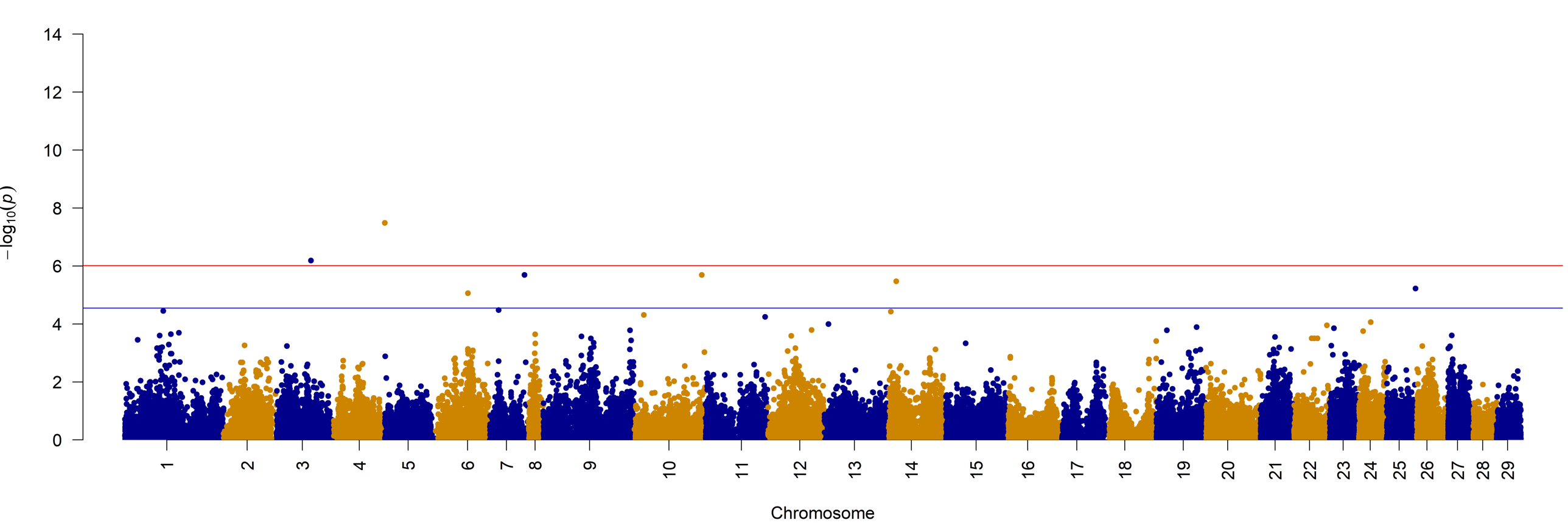


**Figure S8:** Manhattan plot of summary statistics derived from genome-wide association analysis of IGL for YC2018M.


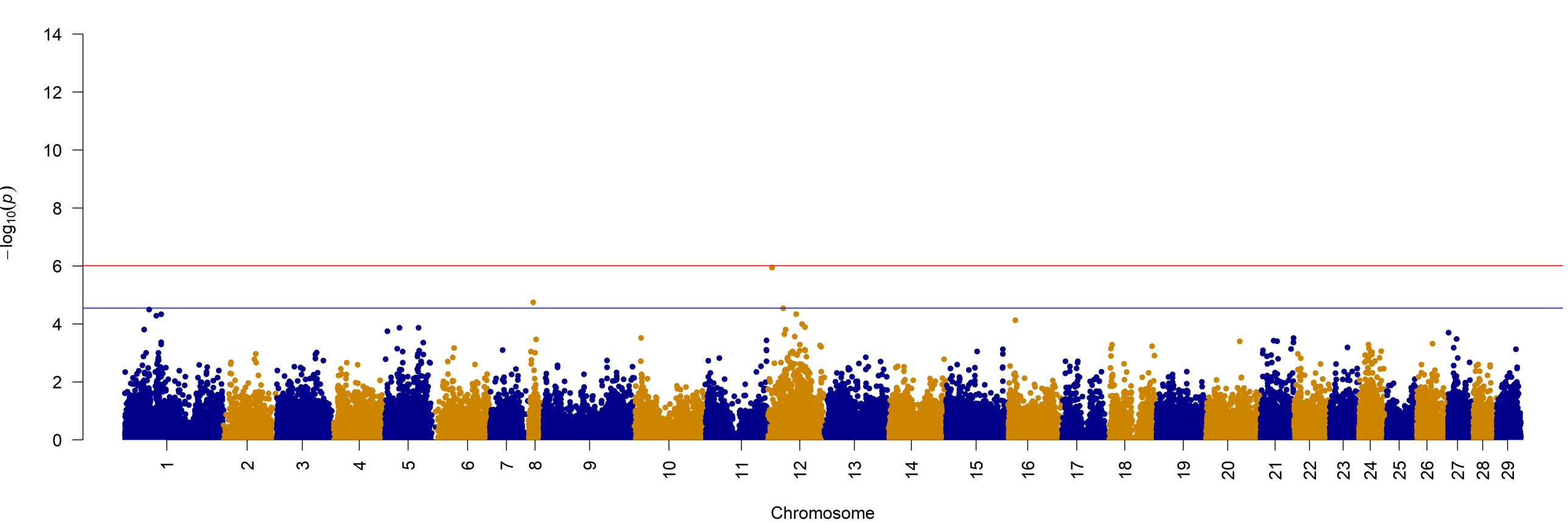


**Figure S9:** Manhattan plot of summary statistics derived from genome-wide association analysis of IGL for YC2018F.


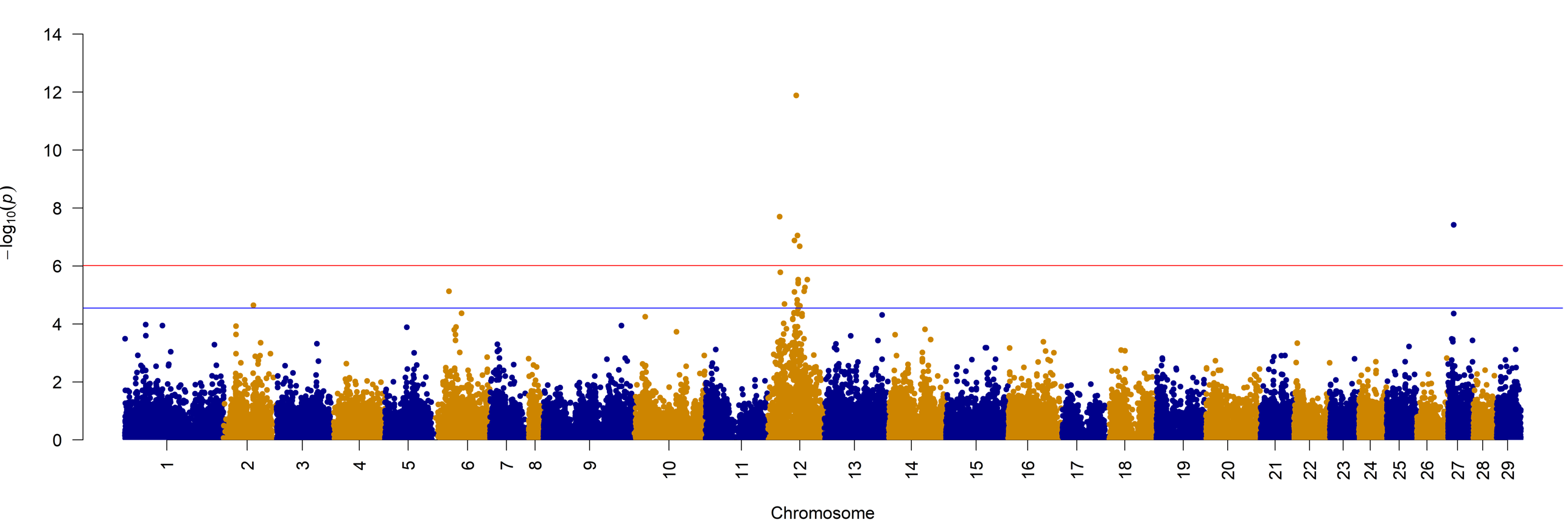


**Figure S10:** Manhattan plot of summary statistics derived from genome-wide association analysis of IGL for YC2018MF.
